# A nuclear-directed human pancreatic ribonuclease (PE5) targets the metabolic phenotype of cancer cells

**DOI:** 10.18632/oncotarget.7579

**Published:** 2016-02-22

**Authors:** Anna Vert, Jessica Castro, Marc Ribó, Antoni Benito, Maria Vilanova

**Affiliations:** ^1^ Laboratori d'Enginyeria de Proteïnes, Departament de Biologia, Facultat de Ciències, Universitat de Girona, Girona, Spain; ^2^ Institut d'Investigació Biomèdica de Girona Josep Trueta, (IdIBGi), Girona, Spain

**Keywords:** antitumor drug, human pancreatic ribonuclease, metabolism of cancer cells, microarray profiling, tumor cell death

## Abstract

Ribonucleases represent a new class of antitumor RNA-damaging drugs. However, many wild-type members of the vertebrate secreted ribonuclease family are not cytotoxic because they are not able to evade the cytosolic ribonuclease inhibitor. We previously engineered the human pancreatic ribonuclease to direct it to the cell nucleus where the inhibitor is not present. The best characterized variant is PE5 that kills cancer cells through apoptosis mediated by the p21^WAF1/CIP1^ induction and the inactivation of JNK. Here, we have used microarray-derived transcriptional profiling to identify PE5 regulated genes on the NCI/ADR-RES ovarian cancer cell line. RT-qPCR analyses have confirmed the expression microarray findings. The results show that PE5 cause pleiotropic effects. Among them, it is remarkable the down-regulation of multiple genes that code for enzymes involved in deregulated metabolic pathways in cancer cells.

## INTRODUCTION

Among the special biological actions exhibited by several members of the vertebrate secreted ribonuclease (RNase) family, selective cytotoxicity for cancer cells is one of the most interesting (for reviews see [1–3]). In contrast to many chemotherapeutic drugs currently used in cancer therapy, which target DNA synthesis and transcription, cytotoxic RNases target RNA functions, such as protein synthesis or gene regulation (for a review see [4]) and, therefore, they are non-mutagenic antitumor drugs.

Although the exact mechanism used by RNases to kill cancer cells is not completely understood, from the knowledge gained so far, a multi-step model has been generally accepted. The model postulates that RNases initially interact with the cell membrane and then internalization proceeds via endocytosis. At some point in the endocytic pathway, cytotoxic RNases are translocated to the cytoplasm where they cleave cellular RNA(s), inhibiting protein synthesis and inducing apoptosis. The cytotoxic action of some RNases may be hampered by the action of a potent inhibitor, the ribonuclease inhibitor (RI) that is found in the cytoplasm of mammalian cells. RI is a 50 kDa protein that tightly binds to some RNases inhibiting their activity [5]. Thus, the cytotoxic potential of a particular RNase also depends on its ability to by-pass the RI action [6].

The members of the vertebrate secreted RNase family that naturally present cytotoxic activity selective for tumor cells evade the RI. Among them, Onconase^®^ (ONC), an amphibian RNase isolated from oocytes of *Rana pipiens*, is the paradigm [3]. However, its clinical use has been limited because it induces renal toxicity at high concentrations [7]. In contrast, mammalian pancreatic RNases accumulate to a much lesser extent in kidneys [8], are less immunogenic, display a higher ribonucleolytic activity [9] but are very sensitive to the RI. To endow them with cytotoxic properties, they have been engineered to either evade the RI [10–12] or to be more efficiently delivered into the cells to ensure a competent arrival to the cytosol to saturate the RI [13,14].

We developed another strategy consisting in routing the RNase into the nucleus. Nuclear compartment, or at least its nucleolus, has been described to be free of RI [15,16]. We designed and produced nuclear-directed human pancreatic RNase (ND-RNase) variants that are cytotoxic for cancer cells [17,18]. Among them, the best characterized is PE5, endowed with a non-contiguous extended bipartite nuclear localization signal (NLS) [19]. This NLS binds specifically to α-importin [20], which drives the RNase into the nucleolus in a GTP- and Ran-dependent manner [17]. Once there, the ND-RNase cleaves nuclear RNA leaving cytoplasmic RNA unaffected [20]. It has been shown that the introduction of this NLS is necessary for the nuclear transport of PE5 and crucial for its cytotoxicity [17,19,20]. *In vitro*, PE5 selectively kills tumor cells. Its cytotoxicity is produced through apoptosis activating caspases-8 and -9 and the executioner caspase-3 [21]. It is noteworthy that PE5 induced apoptosis is associated with the p21^WAF1/CIP1^ induction and the inactivation of JNK. Cell death induced by PE5 also increases the number of cells in S- and G2/M-phases in the NCI/ADR-RES cell line [21]. The cytotoxic effect of PE5 is not prevented by a mutated p53 or a multidrug resistance (MDR) phenotype [21]. Finally, PE5 is synergic with doxorubicin on NCI/ADR-RES cells [22].

To get a deeper insight on the molecular cytotoxic mechanism of PE5, we have used microarray technology to identify significant altered gene expression upon treatment of the NCI/ADR-RES ovarian cancer cell line with PE5. The present work is the first to use microarray-derived transcriptional profiling to identify ND-RNases-regulated genes to understand their cytotoxic effect. It is described that RNases, as RNA-damaging drugs, cause pleiotropic effects [3]. Remarkably, PE5 down-regulates multiple genes that code for enzymes involved in deregulated metabolic pathways in cancer cells. Inhibition of these metabolic processes in cancer cells leads to a significant inhibition of cell growth and induction of cell death. This approach represents a key anticancer strategy that can be used in combination with other antitumor therapies [23–25].

## RESULTS

Since the initial characterization of the cytotoxic mechanism of PE5 was performed in the ovarian cancer cell line, NCI/ADR-RES, we decided to extend the study using microarray technology on the same cell line.

### Cell proliferation and RNase sensitivity

PE5 has ribonucleolytic activity and to get an unbiased approach to examine gene expression by microarray technology, it is very important to search for optimal cell treatment conditions. The RNA population has to be representative of the actual drug effect and not the result of an extensive RNA degradation that would preclude the chance of microarray analysis. In addition, because the induction of apoptosis increases the RNA turnover [26], an extensive cytotoxic effect would generate changes in the RNA levels as a consequence of cellular apoptosis rather than the direct action of the RNase. Therefore, we investigated the cytotoxic effect of PE5 on NCI/ADR-RES cell line at different RNase concentrations and incubation times (Figure S1, Supplementary Data). A dose-dependent cell growth inhibition was apparent after 36 h of incubation with PE5 that increased with time.

Then we analyzed the RNA degradation caused by the different treatments. We incubated NCI/ADR-RES cells with PE5 concentrations that induced a decrease of cell proliferation of 5%, 10%, and 15% (IC_5_, IC_10_, and IC_15_) after 36 h of incubation. We did not analyze higher RNase concentrations because we had previously observed an extensive cleavage of nuclear RNA in HeLa cells incubated with PE5 at a concentration equivalent to IC_20_ [20]. The RNA degradation of treated and untreated cells was quantified using a bioanalyzer. The RNA integrity obtained after PE5 treatment at the three concentrations, IC_5_, IC_10_, and IC_15_ gave RIN values of 8.8, 7.9 and 6.60, respectively. Accordingly, we carried out the microarray experiments at a PE5 concentration of 12 μM that triggered a decrease of 10% of cell viability, after 36 h of treatment, without an extensive RNA degradation.

### Gene expression changes in PE5-treated cells

PE5-treated and untreated NCI/ADR-RES cells revealed 647 differentially expressed genes out of 35,377 present in the microarray (1.83%). Among them, 47% were down-regulated (decrease from untreated cells ranged from 2- to 4-fold) while 53% were up-regulated in PE5-treated cells (increase from untreated cells ranged from 2- to 106-fold). This suggests that the primary effect of PE5 is both to increase and to decrease gene expression. Among the PE5 down-regulated genes many are known to be involved in cell adhesion and migration (e.g. GPC6, and EFEMP1), amino acid metabolism (e.g. PYCR1, BCAT1, PHGDH and ASNS), lipid metabolism (e.g. HADHA, DHCR24, ACACA and SPTLC3), and glucose metabolism (e.g. PGM1, PGAM1, LDHA and ENO1). In particular, some of them are known to be up-regulated in tumor cells enhancing the aggressive nature of tumors (e.g. EFEMP1), promoting migration and invasion of cancer cells (e.g. GPC6) or acting as proto-oncogenes (e.g. MET). Among the PE5 up-regulated genes many are known to be involved in transcription regulation (e.g. HMBOX1, SPEN, RPA4 and MXD1), apoptosis (e.g. BCL2L11, WWOX and BNIP3L), and signaling pathways or transduction (e.g. LRRC2, PPP6R1, RAP1GAP and CISH). Interestingly, some of them function as tumor suppressors (e.g. BCL2L11, MXD1, WWOX, BNIP3L, and DMTF1) or are candidate tumor suppressors having anti-proliferative properties and DNA repair abilities (e.g. LRRC2, RPA4, PPP6R1, SPEN, RAP1GAP and CISH) (see discussion section). Some of the above genes are among the top 20 PE5 up- and down-regulated genes in NCI/ADR-RES cell line (Table S1, Supplementary Data).

### RT-qPCR analysis of gene expression

mRNA expression of six PE5 down-regulated genes (G6PD, ACACA, PHGDH, IDH2, AKR1A1, and MET) and one up-regulated gene (BCL2L11), representatives of the processes affected by this RNase, were examined by RT-qPCR. The results are presented in Figure S2 of Supplementary Data. Fold changes obtained with RT-qPCR, calculated as the ratio between relative transcript abundance (RTA) values obtained for PE5 treated cells and RTA values obtained for untreated cells, were similar to those found in the microarray analysis (Table 1) confirming that the microarray experiments are fully valid.

**Table 1:** Comparison of fold change values obtained by RT-qPCR and gene expression microarray experiments from PE5-treated cells respective to untreated cells

Gene Symbol	Gene Name	Fold Change RT-qPCR ^a^	Fold Change microarrays
G6PD	Glucose-6-phosphate dehydrogenase	−2.18 ± 0.41	−2.20
ACACA	Acetyl-CoA carboxylase alpha	−2.69 ± 0.59	−2.27
PHGDH	Phosphoglycerate dehydrogenase	−3.53 ± 0.74	−2.71
IDH2	Isocitrate dehydrogenase 2 (NADP+), mitochondrial	−2.21 ± 0.34	−2.33
AKR1A1	Aldo-keto reductase family 1, member A1 (aldehyde reductase)	−2.00 ± 0.24	−2.28
MET	Met proto-oncogene (hepatocyte growth factor receptor)	−3.00 ± 0.65	−2.60
BCL2L11	BCL2-like 11 (apoptosis facilitator)	2.13 ± 0.25	3.19

a Mean ± SD

### Gene ontology analysis and KEGG pathway annotation

Although the information obtained from individual gene expression changes as a response to PE5 treatment gives clues to the action of this RNase, to better understand the functional relevance of its regulated genes in NCI/ADR-RES cells, we performed a gene ontology analysis. PE5 differentially expressed genes were used to find over-represented gene ontology terms in the three broad ontology categories: “molecular function”, that captures the knowledge about the functional activity of gene products, the larger “biological process” as part of which these specific functions collectively act, and “cellular component” where all this occurs. To the same end, PE5 differentially expressed genes were mapped to KEGG database to find overrepresented known metabolic and regulatory pathways. In all cases, a p-value ≤ 0.05 was considered statistically significant.

Gene ontology analyses showed that PE5 differentially expressed genes are related to different cellular events (Table 2). Among them, we can highlight the biological process ontologies related to lipid and carbohydrate metabolism, response to stress, cell adhesion, proliferation and migration. Interestingly, response to stress term includes genes involved in reactive oxygen species (ROS) quenching and antitumor therapy resistance. Likewise, cell proliferation contains genes that act as tumor suppressors or oncogenes. The individual function of PE5 differentially expressed genes favored nucleotide binding, peptidase activity, and pyrophosphatase activity and they were mainly associated with lysosomes, plasma membrane and anchoring junction.

**Table 2:** Gene ontology analysis of PE5 differentially expressed genes in NCI/ADR-RES cell line

Gene Ontology	Gene ontology term	Gene count ^a^	% Down-regulated genes	P-value
Biological Process	Lipid metabolic process	64	73.44	8.11E-05
	Response to stress	131	63.36	4.70E-04
	Angiogenesis	25	64.00	6.83E-04
	Developmental process	181	61.88	4.07E-03
	Cell adhesion	47	65.96	8.26E-03
	Cell proliferation	69	49.28	1.33E-02
	Cell migration	41	78.05	1.61E-02
	Carbohydrate metabolic process	36	69.44	4.34E-02
Molecular Function	Nucleotide binding	115	60.00	3.52E-05
	Peptidase activity	34	79.41	1.34E-03
	Pyrophosphatase activity	42	50.00	2.58E-03
	Oxidoreductase activity	38	68.42	3.30E-03
	Monosaccharide binding	7	100.00	4.58E-03
	Cytoskeletal protein binding	34	64.71	4.95E-03
	Glycoprotein binding	6	66.67	1.28E-02
	Coenzyme binding	12	75.00	1.77E-02
Cellular Component	Lysosome	31	90.32	2.31E-07
	Plasma membrane	177	64.97	1.27E-04
	Anchoring junction	16	81.25	1.67E-03
	Endoplasmic reticulum	61	73.77	2.25E-03
	Cell surface	28	78.57	2.41E-03
	Extracellular region	92	73.91	3.05E-03
	Endosome	29	58.62	7.86E-03
	Golgi apparatus	53	73.58	8.01E-03
	Actin cytoskeleton	19	68.42	2.87E-02

a Number of differentially expressed genes that belong to these terms.

Analysis of the over-represented pathways collected in the KEGG database showed that 19 pathways were affected by PE5 treatment (Table 3). Most of them were involved in cell metabolism and, of particular interest, are those of pyruvate and glucose metabolism for their relevance in cancer cells [27].

**Table 3:** KEGG pathway annotation of PE5 differentially expressed genes in NCI/ADR-RES cell line

KEGG term	Gene count ^a^	% Down-regulated genes	P-value
Lysosome	14	92.86	8.84E-05
Metabolic pathways	59	88.14	8.73E-04
Pyruvate metabolism	6	66.67	2.64E-03
Steroid biosynthesis	4	100.00	3.97E-03
Propanoate metabolism	5	80.00	5.01E-03
Glycolysis / Gluconeogenesis	7	85.71	7.91E-03
Nicotinate and nicotinamide metabolism	4	100.00	9.47E-03
Gastric acid secretion	7	71.43	1.56E-02
Homologous recombination	4	25.00	1.63E-02
Vitamin B6 metabolism	2	100.00	1.72E-02
Selenocompound metabolism	3	66.67	2.09E-02
Other glycan degradation	3	100.00	2.09E-02
Aminoacyl-tRNA biosynthesis	6	100.00	2.38E-02
Focal adhesion	13	92.31	2.55E-02
Glycosaminoglycan degradation	3	66.67	2.83E-02
ECM-receptor interaction	7	100.00	3.10E-02
Viral myocarditis	6	66.67	3.75E-02
Salivary secretion	7	85.71	3.85E-02
Bile secretion	6	50.00	3.98E-02

a Number of differentially expressed genes that belong to these terms.

## DISCUSSION

Gene ontology analysis clustered the 647 PE5 affected genes to different terms (Table 2). Among them, we have considered that lipid and carbohydrate metabolism, response to stress and cell adhesion and proliferation are the most relevant to understand how PE5 arrests NC/ADR-RES cell proliferation and induces apoptosis. Moreover, KEGG analysis shows that 11 out of 19 terms are related to metabolic processes (Table 3). Most of the genes involved in these terms are down-regulated. Although they constitute a 47% of the total affected genes, this percentage significantly increases when considering the genes belonging to the above mentioned terms (Tables 2 and 3). It is tempting to speculate that the expression of up-regulated genes could be due to the cleavage of different microRNAs, as it has been demonstrated for ONC [28]. Further, whereas PE5 down-regulated genes can be clustered in gene ontology and KEGG terms to explain PE5 cytotoxic action, the PE5 up-regulated genes, with the exception of tumor suppressors, cannot. Interestingly, among the 20 more up-regulated genes (Table S1 Supplementary Data), six are candidate tumor suppressors (see below). Thus, although we cannot rule out a role of PE5 up-regulated genes in the cytotoxic mechanism of this drug most of the genes discussed below are down-regulated.

### PE5 down-regulates genes coding for enzymes involved in deregulated metabolic pathways in cancer cells

**Figure 1:**
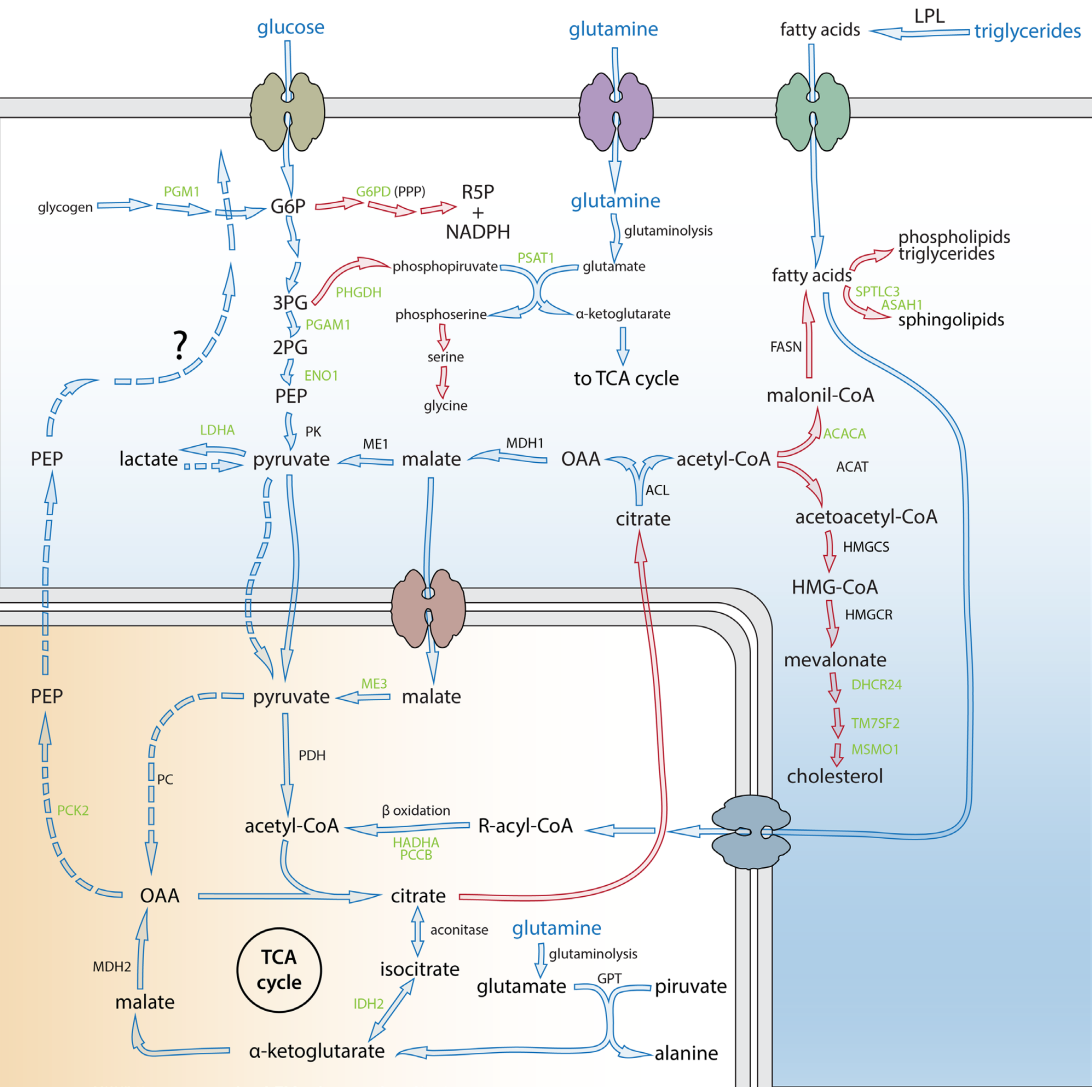
PE5 down-regulated enzymes belonging to deregulated metabolic pathways in cancer Green, name of the enzymes down-regulated by PE5; blue, metabolites that are the main source of energy for cancer cells depending on their status; blue arrows, catabolic pathways; blue dashed arrows, potential gluconeogenesis not still proved in cancer cells (see text for more details); red arrows, biosynthetic pathways critical for cancer cell grow and division; PPP, pentose phosphate pathway; TCA, tricarboxylic acid; G6P, glucose-6-phosphate; OAA, oxaloacetate; PEP, phosphoenolpyruvate; 2PG, 2-phosphoglycerate; 3PG, 3-phosphoglycerate; HMG-CoA, 3-hydroxy-3-methylglutaryl-coenzyme A; R5P, ribose-5-phosphate. Enzymes: ACACA, acetyl-CoA carboxylase alpha; ACAT, acetyl-CoA acetyltransferase; ACL, ATP citrate lyase; ASAH1, N-acylsphingosine amidohydrolase (acid ceramidase) 1; DHCR24, 24-dehydrocholesterol reductase; ENO1, enolase 1, (alpha); FASN, fatty acid synthase; GPT, alanine aminotransferase; G6PD, glucose-6-phosphate dehydrogenase; HADHA, hydroxyacyl-CoA dehydrogenase/3-ketoacyl-CoA thiolase/enoyl-CoA hydratase (trifunctional protein), alpha subunit; HMGCR, HMG-CoA reductase; HMGCS, HMG-CoA synthase; IDH2, isocitrate dehydrogenase 2 (NADP+), mitochondrial; LDHA, lactate dehydrogenase A; LPL, lipoprotein lipase; MDH1, malate dehydrogenase 1, NAD (soluble); MDH2, malate dehydrogenase 2, NAD (mitochondrial); ME1, malic enzyme 1, NADP+-dependent, cytosolic; ME3, malic enzyme 3, NADP+-dependent, mitochondrial; MSMO1, methylsterol monooxygenase 1; PC, pyruvate carboxylase; PCCB, propionyl CoA carboxylase, beta polypeptide; PCK2, phophoenolpyruvate carboxykinase 2 (mitochondrial); PDH, pyruvate dehydrogenase complex; PGAM1, phosphoglycerate mutase 1 (brain); PGM1, phosphoglucomutase 1, PHGDH, phosphoglycerate dehydrogenase; PK, pyruvate kinase; PSAT1, phosphoserine aminotransferase 1; SPTLC3, serine palmitoyltransferase, long chain base subunit 3; TM7SF2, transmembrane 7 superfamily member 2.

PE5 down-regulates genes related to glucose metabolism such as phosphoglycerate mutase 1 (**PGAM1**), enolase 1, (alpha) (**ENO1**), glucose-6-phosphate dehydrogenase (**G6PD**), phosphoglycerate dehydrogenase (**PHGDH**), phosphoserine aminotransferase (**PSAT1**) and phosphoglucomutase 1 (**PGM1**). PGAM1 and ENO1 repression produce a decrease in the glycolytic flux associated with a reduction of tumor growth [29–32]. Both genes are over-expressed in different types of cancer [31–34]. G6PD derives the G6P from glycolysis to the pentose phosphate pathway (PPP) (Figure 1), a critical way to obtain both ribose-5-phosphate for biosynthesis and reducing equivalents in form of NADPH, which are used not only for biosynthesis but for scavenging cellular ROS [35]. PHGDH and PSAT1 are critical to derive a significant portion of glycolytic carbon to the biosynthesis of serine and glycine amino acids (Figure 1). PHGDH has been identified as a gene frequently amplified in different cancers [36,37]. PGM1 is an enzyme that can increase the levels of G6P through glycogenolysis. It has been described that glycogen-derived glucose contributes to ATP generation without oxygen requirement, ROS scavenging, and biosynthesis of macromolecules [38].

PE5 down-regulates the gene coding for lactate dehydrogenase A (**LDHA**). The acidification of tumor surroundings produced by excreted lactate helps tumor invasion and inhibits the immune system. Moreover, lactate is a gluconeogenic precursor and although the role of gluconeogenesis in cancer is still not well known, the key mitochondrial gluconeogenic enzyme, phophoenolpyruvate carboxykinase 2 mitochondrial (**PCK2**), also down-regulated by PE5, is expressed in different non-gluconeogenic tissues including tumors [39–41]. Recently [42] it has been demonstrated that in lung cancer cell lines incubated with ^13^C-lactate, the three lactate carbon atoms appear in the phosphoenolpyruvate (PEP) pool, supporting a conversion of lactate to PEP via gluconeogenesis (Figure 1). This suggests that some steps of gluconeogenesis are used to overcome the detrimental metabolic situation generated by aglycemia periods generated by the sustained high rate of cell proliferation [43].

PE5 down-regulates some enzymes involved in the oxidation of fatty acids, a key pathway for energy generation in cancer cells [44]. We can mention propionyl CoA carboxylase beta polypeptide (**PCCB)**, as well as hydroxyacyl-CoA dehydrogenase/3-ketoacyl-CoA thiolase/enoyl-CoA hydratase (trifunctional protein), alpha subunit (**HADHA)** (Figure 1).

On the other hand, cancer cells are highly dependent on *de novo* lipid biosynthesis [44]. PE5 down-regulates acetyl-CoA carboxylase, alpha (**ACACA**) (Figure 1). Citrate is a critical metabolite required to support cytosolic lipid biosynthesis. In cancer cells, TCA cycle anaplerosis is maintained mainly by glutamine [45,46]. Glutamine-derived α-ketoglutarate is reductively carboxylated by isocitrate dehydrogenase 1 or 2 (IDH1, IDH2) to isocitrate/citrate (Figure 1) [47,48]. NADPH-linked mitochondrial isocitrate dehydrogenase 2 (**IDH2**) is a PE5-down-regulated enzyme. Interestingly, it has recently described that IDH2 is involved in the generation of oncometabolite 2-hydroxiglutarate (2-HG) [49]. Cells have other ways to refurbish TCA cycle [45]. Mitochondrial extruded citrate converted to OAA and acetil-CoA by ATP citrate lyase (ACL) can re-enter the OAA moiety through several steps (Figure 1) that include the PE5 down-regulated NADP^+^-dependentmitochondrial malic enzyme 3 (**ME3**).

The increased fatty acid synthesis leads to the up-regulation of the phospholipids [50], sphingolipids [51] and cholesterol biosynthesis [44]. Some genes involved in sphingolipid synthesis have a decreased expression upon PE5 cell treatment: serine palmitoyltransferase long chain base subunit 3 (**SPTLC3**) and N-acylsphingosine amidohydrolase (acid ceramidase) 1 (**ASAH1**). PE5 also down-regulates some key enzymes involved in cholesterol synthesis such as 24-dehydrocholesterol reductase (**DHCR24)**, transmembrane 7 superfamily member 2 (**TM7SF2),** monooxygenase 1 (**MSMO1)** (Figure 1). It is worth mentioning that deregulation of the mevalonate pathway has been associated with transformation [52–54].

Although we have not found a term in gene ontology and KEGG analysis related to amino acid metabolism it is worth mentioning that PE5 treatment reduces the expression level of genes involved in amino acid biosynthesis other than PHGDH and G6PD described above. These genes are pyrroline-5-carboxylate reductase 1 (**PYCR1**), asparagine synthetase (**ASNS**), and the catabolizing amino acid enzyme, branched-chain amino acid transaminase 1 (**BCAT1**). All three enzymes are found over-expressed in different cancers and ASNS is associated with resistance to L-asparaginase cancer therapy [55–57]. Interestingly, PHGDH, PYCR1 and BCAT1 are among the 20 most PE5 down-regulated genes (Table S1 Supplementary Data).

KEGG analysis shows that PE5 may also inhibit the protein synthesis since it down-regulates many genes coding for aminoacyl tRNA synthetases. These genes are cysteinyl-tRNA synthetase (**CARS**), alanyl-tRNA synthetase (**AARS**), glycyl-tRNA synthetase (**GARS**), isoleucyl-tRNA synthetase (**IARS**), tyrosyl-tRNA synthetase (**YARS**), and glutamyl-prolyl-tRNA synthetase (**EPRS**). This is in agreement with our previous results that showed that treatment of different cancer cell lines with PE5 reduces cell protein synthesis compared to untreated cells [17].

### PE5 down or up-regulates some oncogenes and tumor suppressor genes, respectively

Among the genes with oncogenic functions down-regulated by PE5, we can mention glypican 6 (**GPC6**), EGF containing fibulin-like extracellular matrix protein 1 (**EFEMP1**), met proto-oncogene (hepatocyte growth factor receptor) (**MET**), transglutaminase 2 (C polypeptide, protein-glutamine-gamma-glutamyltransferase) (**TGM2**), platelet-derived growth factor receptor, beta polypeptide (**PDGFRB**), and clusterin (**CLU**). All of them have been found overexpressed in different tumors where they play different roles ranging from cell proliferation and angiogenic stimulation to invasiveness and metastasis [58–67].

**Figure 2:**
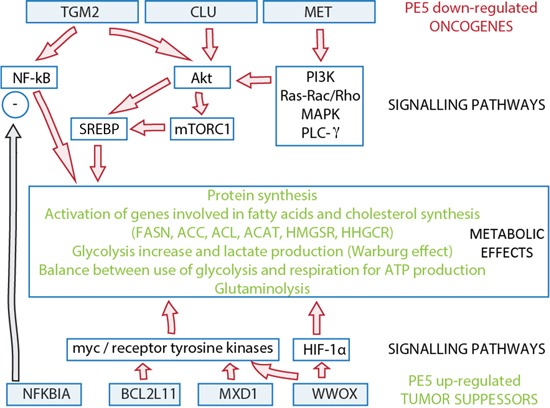
Potential links between PE5 down-regulated oncogenes and up-regulated tumor suppressors and deregulated metabolic pathways through signaling pathways The PE5 down-regulated oncogenes and the PE5 up-regulated tumor suppressors are shown in the blue boxes while the potential signaling pathways are indicated in the empty boxes. The deregulated metabolic pathways are inhibited by both the down-regulation of the oncogenes and the up-regulation of the tumor suppressor (see text for more details). Genes: BCL2L11, BCL2-like 11 (apoptosis facilitator); CLU, clusterin; MET, met proto-oncogene (hepatocyte growth factor receptor); MXD1, MAX dimerization protein 1; NFKBIA, nuclear factor of kappa light polypeptide gene enhancer in B-cells inhibitor, alpha; TGM2, transglutaminase 2 (C polypeptide, protein-glutamine-gamma-glutamyltransferase); WWOX, WW domain containing oxidoreductase.

Interestingly, MET, TGM2 and CLU are linked to some deregulated metabolic pathways, inhibited by PE5, through the activation of signaling pathways (Figure 2). The binding of MET with its ligand (hepatocyte growth factor) activates downstream signaling pathways, including phosphoinositide 3-kinase (PI3K)/Akt, Ras-Rac/Rho, MAPK, and phospholipase C-γ [64], frequently activated in human cancers [68]. TGM2 activates the pro-survival NF-κB [69] and focal adhesion kinase/Akt, whereas it negatively regulates the tumor suppressor phosphatase and tensin homologue (PTEN) [70]. PTEN suppression in malignant cells increases the PI3K system activity [27]. CLU activates the pro-survival Akt [71] while inhibiting the pro-apoptotic Bax [72]. Akt can activate ACL by phosphorylation [73] and activate the expression of several genes involved in fatty acid and cholesterol biosynthesis, such as FASN, ACC, ACL, ACAT, HMGCS and HMGCR, through their effects on the transcription factor family of sterol regulatory element-binding proteins (SREBPs) [74]. One important downstream effector of Akt is the mammalian target of rapamycin complex I (mTORC1), involved in the regulation of several metabolic processes, including protein synthesis [75], whose activity is required for the nuclear accumulation of mature SREBPs. Activation of PI3K/Akt/mTOR pathway also increases glycolysis and lactate production and is sufficient to induce the Warburg effect [27]. It is also worth mentioning that GPC6, the most PE5 down-regulated gene, belongs to the glypican family of cell surface heparan sulfate proteoglycans [76]. Expression of GPC6 promotes invasive migration through up-regulation of Wnt5A signaling. More interesting, GPC6 induction of Wnt5A stimulates the activation of JNK and p38 MAPK [77]. We have demonstrated that PE5 kills cancer cells through apoptosis associated with the p21^WAF1/CIP1^ induction and JNK inactivation [21], thus a PE5 down-regulation of GPC6 might contribute to JNK inactivation.

Tumor suppressor genes up-regulated by PE5 treatment are the apoptosis facilitator BCL2-like 11 (**BCL2L11**), MAX dimerization protein 1 (**MXD1**), ras homolog gene family, member B (**RHOB**), BCL2/adenovirus E1B 19kDa interacting protein 3-like (**BNIP3L**), cyclin D binding myb-like transcription factor 1 (**DMTF1**), WW domain containing oxidoreductase (**WWOX**), and nuclear factor of kappa light polypeptide gene enhancer in B-cells inhibitor, alpha (**NFKBIA**). All of them play two main roles, apoptosis activation or facilitation [78–81] and oncogene antagonism [82–86]. In addition, among the 20 most PE5 up-regulated genes (Table S1, Supplementary Data) some are candidate tumor suppressors: Leucine rich repeat containing 2 (**LRRC2**) [87,88], Replication protein A4, 30kDa (**RPA4**) [89,90], Protein phosphatase 6, regulatory subunit (**PPP6R1**) [91,92], Spen homolog, transcriptional regulator (*Drosophila*) (**SPEN**) [93], RAP1GTPase activating protein (**RAP1GAP)** [94] and cytokine inducible SH2-containing protein (**CISH**) [95].

Like oncogenes, some of these tumor suppressor genes are related to metabolic reprogramming of cancer cells (Figure 2). BCL2L11 interacts with all anti-apoptotic members of the Bcl-2 protein family and acts as an apoptotic activator [78]. Loss of even one BCL2L11 allele accelerates Myc-induced development of tumors [96]. MXD1 is considered an antagonist of the oncogene Myc [82]. WWOX interacts with different oncogenic proteins sequestering them in the cytoplasm and thereby inhibiting their oncogenic activity [97–99]. Several oncogenes, like Myc and receptor tyrosine kinases can stimulate the transcription of a number of genes encoding the proteins that mediate glycolysis and glutaminolysis pathways [100]. Moreover, WWOX controls the expression of glycolytic genes through hypoxia-inducible transcription factor-1α regulation (HIF-1α) [101–103]. Therefore, PE5-induced over-expression of BCL2L11, MXD1 and WWOX reinforces the suppression of the metabolic reprograming of cancer cells induced by PE5 down-regulated oncogenes described above. NFKBIA inhibits the transcription factor NF-kB [86]. Thus, PE5 can reduce the NF-kB activity both by down-regulating its activator oncogene (TGM2) and by up-regulating its inhibitor (NFKBIA). The NF-kB pathway has been recently described as involved in metabolic reprogramming [104].

### PE5 down-regulates genes related to ROS quenching and antitumor therapy resistance

In addition to the effects mentioned above, PE5 decreases the expression of genes involved in the quenching of ROS. Treatment with PE5 reduces the expression of two genes involved in the detoxification of ROS, thioredoxin reductase 2 (**TXNRD2**) and glutathione peroxidase 3 (**GPX3**), belonging to thioredoxin [105] and glutathione antioxidant systems [106], respectively. Of note, glutathione and thioredoxin antioxidant systems rely on the reducing power of NADPH to maintain their activities. As stated above, PE5 decreases the expression level of relevant proteins involved in the production of NADPH (G6PD, ME3 and IDH2). Altogether, this reduction can induce apoptosis due to high ROS levels in cancer cells treated with PE5.

On the other hand, reduced glutathione has an important role in a number of drug resistance mechanisms [107]. In addition, some of the above mentioned PE5 down-regulated genes are involved in resistance to antitumor therapy. For instance, **MET** whose activation is related to resistance to epidermal growth factor receptor inhibitors [108], **PDGFRB** whose inhibition is associated with an improved tumor drug uptake [109], **CLU** that interferes with apoptotic signaling and confers resistance to a broad spectrum of anti-cancer treatments [110,111], and **ASNS** involved in resistance to L-asparaginase [56,112]. Likewise, up-regulation of some of the above stated tumor suppressors such as BCL2L11, RHOB and NFKBIA, contributes to fight against the antitumor drug resistance because they mediate apoptosis induced by several anticancer drugs [113–115]. PE5 also down-regulates the expression of two enzymes directly involved in the MDR phenotype: i) aldo-keto reductase family 1 member A1 (**AKR1A1**) that metabolizes anthracyclines into inactive compounds [116] and has been associated with acquired resistance to irradiation [117]; ii) prosaposin (**PSAP**), present among the 20 most PE5 down-regulated genes (Table S1 Supplementary Data), found overexpressed in ovarian tumors after chemotherapy [118] and involved in estrogen chemotherapy resistance [119].

## MATERIALS AND METHODS

### PE5 expression and purification

Construction of PE5 has been previously described [17]. It was obtained from PM5 (a human pancreatic RNase variant carrying five substitutions at the N-terminus: Arg4Ala, Lys6Ala, Gln9Glu, Asp16Gly, and Ser17Asn [120]) by replacing Gly89 and Ser90 by Arg. PE5 was produced and purified from *Escherichia coli* BL21(DE3) cells transformed with the corresponding vector essentially as described previously [121]. The molecular mass of PE5 was confirmed by Matrix-assisted laser desorption/ionization time-of-flight (MALDI-TOF) mass spectrometry at *Unitat cientificotècnica de suport*, *Institut de Recerca*, *Hospital Universitari Vall d'Hebron* (Barcelona, Spain). PE5 concentration was determined by ultraviolet spectroscopy using an extinction coefficient at 280 nm of 7950 M^−1^ cm^−1^, calculated as reported previously [122].

### Cell line and culture conditions

NCI/ADR-RES human ovarian cancer MDR cell line (formerly MCF-7/AdrR) [123] was a generous gift of Dr. Ramon Colomer of the *Institut Català d'Oncologia de Girona*, *Hospital Universitari de Girona Dr. Josep Trueta* (Girona, Spain). They were initially obtained from American type Culture Collection (ATCC) (Manassas, Virginia) and were used immediately after resuscitation. Cells were routinely grown at 37°C in a humidified atmosphere of 5% CO_2_ in DMEM (Gibco, Germany) supplemented with 10% fetal bovine serum (Gibco, Germany), 50 U/ml penicillin, 50 μg/ml streptomycin (Gibco, Germany), and 1.84 μM doxorubicin (Tedec-Meijic Farma, Spain). Cells remained free of *Mycoplasma* and were propagated according to established protocols.

### Cell proliferation assay

NCI/ADR-RES cells (10^4^ per well) were seeded into 96-well plates. After 24 h of incubation, cells were treated for 24, 36, or 48 h with various concentrations of PE5, ranging from 0.1 to 30 μM. RNase sensitivity was determined by the MTT method essentially as described by the manufacturer's instructions (Sigma, USA). Data were collected by measuring the absorbance at 570 nm with a Synergy 4 multi-well plate reader (Biotek Instruments, USA). The IC_5_, IC_10_, and IC_15_ values represent the concentrations of PE5 required to inhibit cell proliferation by 5, 10, and 15%, respectively, and were calculated by interpolation from the obtained growth curves. Data are expressed as mean ± SD of three independent experiments conducted in triplicates.

### PE5 treatment and RNA isolation

NCI/ADR-RES cells (2×10^5^ per well) were seeded into 6-well plates. After 24 h of incubation, cells were treated for 36 h with a concentration of PE5 that caused a 10% decrease of cell proliferation (12 μM). Cells were then harvested at 400 xg for 5 min at 4°C and washed twice with cold PBS. Total RNA was extracted using the mirVana miRNA isolation kit (Applied Biosystems/Ambion, USA) according to the manufacturer's instructions and stored at −80°C. Four independent preparations were performed. RNA integrity and absorbance 260/280 nm ratio of each sample were checked using an Agilent 2100 Bioanalyzer (Agilent Technologies, USA) and a NanoDrop ND-1000 Spectrophotometer (Thermo Fisher Scientific, USA), respectively.

### Gene expression microarray analysis

Gene expression microarray experiments were performed at *Bioarray, S.L*. (Alacant, Spain) using the SurePrint G3 Human Gene Expression Microarray (Agilent Technologies, USA), a high-density oligonucleotide microarray that contains 60,000 probes that correspond to 27,958 Entrez Gene RNAs and 7,419 lncRNAs. Sample preparation and microarray processing procedures were done according to the Two-Color Microarray-Based Gene Expression Analysis v. 6.5 (Agilent Technologies, USA). Briefly, 200 ng of total RNA were used to synthesize double-stranded cDNA with AffinityScript-Reverse Transcriptase and Oligo dT-Promoter Primer. cDNA was simultaneously amplified and transcribed into cyanine 3- or cyanine 5-labeled cRNA employing T7 RNA Polymerase in presence of cyanine 3-CTP or cyanine 5-CTP. The labeled cRNA (antisense) was purified, evaluated using a NanoDrop ND-1000 Spectrophotometer (Thermo Fisher Scientific, USA) and hybridized to the oligonucleotide microarrays at 65°C for 17 h. Microarrays were then washed and scanned on a G2565CA Microarray Scanner updated to 2 micron resolution (Agilent Technologies, USA). Data were extracted from the resulting TIFF-images using the Feature Extraction software v. 10.7 (Agilent Technologies, USA). Raw microarray data were statistically analysed using the software packages Marray, pcaMethods, Limma, and RankProd from Bioconductor (www.bioconductor.org), which uses the R statistical environment and programming language. In particular, the non-specific signal was removed from the total intensity using the Normexp background correction method with an offset of 20 [124]. Then intra-slide normalization was done using the Loess method [125] to make intensities consistent within each array, and inter-slide normalization was performed employing the Aquantiles method [126] to achieve consistency between arrays. After each of these analyses, a quality control analysis of microarray data (RG density plot, MA plot and M boxplot) was performed. Following normalization, the RankProd method [127] was applied to identify differentially expressed genes. Genes were considered differentially expressed when they had a false discovery rate adjusted p-value ≤ 0.05 and a fold change ≥ 2 or ≤ −2.

Data have been deposited in NCBI's Gene Expression Omnibus repository [128] (http://www.ncbi.nih.gov/geo) and are available under the accession number: GSE75494.

### Gene ontology analysis and KEGG pathway annotation

Differentially expressed genes were characterized functionally with an hypergeometric test to find over-represented gene ontology terms in the three main broad ontologies (biological process, molecular function, and cellular component) (www.geneontology.org), and were also mapped to the Kyoto Encyclopedia of Genes and Genomes (KEGG) (www.kegg.jp), which assigns proteins to pathways, to find over-represented pathways. The analyses were done using the software packages GOstats and RamiGO from Bioconductor (www.bioconductor.org). A p-value cutoff of 0.05 was used.

### Quantitative reverse transcription PCR (RT-qPCR)

mRNA expression of six PE5 down-regulated genes (G6PD, ACACA, PHGDH, IDH2, AKR1A1, and MET) and one up-regulated gene (BCL2L11) were examined by quantitative real-time PCR. The same RNA samples used for microarrays analysis were used for performing this analysis. First, RNA samples were digested with DNase to prevent genomic contamination using the RNase-Free DNase Set (Qiagen, Germany) according to the manufacturer's instructions, and were evaluated using an Agilent 2100 Bioanalyzer (Agilent Technologies, USA) and a NanoDrop ND-1000 Spectrophotometer (Thermo Fisher Scientific, USA). Then, for each sample, 0.5 μg of RNA were used to synthetize single-stranded cDNA with the High-Capacity cDNA Reverse Transcription Kit (Applied Biosystems, USA) following the manufacturer's instructions. Gene-specific forward and reverse primers for the selected genes were designed with Primer3 (http://primer3.ut.ee) and checked with NetPrimer (http://www.premierbiosoft.com/netprimer/). Primer sequences are indicated in Tables S2 and S3 of Supplementary Data for constitutive and target genes, respectively. Real-time PCRs were performed in an optical 96-well plate with an ABI PRISM 7300 Sequence Detector System (Applied Biosystems, USA), using SYBR Green to monitor dscDNA synthesis. Reactions contained 1x Power SYBR Green PCR Master Mix (Applied Biosystems, USA), 300 nM of gene-specific forward primer, 300 nM of gene-specific reverse primer, and 5μl of a 50-fold dilution of the previously synthesized cDNA in a final volume of 20 μl. The following standard thermal profile was used for all real-time PCRs: 95°C for 10 min, 40 cycles of 95°C for 15 s and 60°C for 1 min. A dissociation step was performed after amplification to confirm the presence of a single amplicon. To estimate variation in the technique, three technical replicates were carried out for each cDNA sample. Data were analyzed with the 7300 SDS 1.3.1 software (Applied Biosystems, USA). To generate a baseline-subtracted plot of the logarithmic increase in fluorescence signal (ΔRn) versus cycle number, baseline data were collected between cycles 3 and 15. All amplification plots were analyzed with an Rn threshold of 0.2 to obtain threshold cycle (Ct) values. The amplification efficiency for each gene was calculated based on five dilutions of cDNA ranging from 1 to 3,2×10^−4^ and the equation E = 10^(−1/slope)^. All genes had an efficiency value between 1.85 and 2.05. To select a constitutive gene as a reference for normalizing data, the transcription abundances of five genes (ACTB, GUSB, TBP, HPRT1, and ALAS1) were measured for all cDNA samples. Among them, TBP showed the highest stability (lower standard deviation of the Ct) (Figure S3, Supplementary Data) and therefore was selected as the reference gene for normalizing data. For the target genes (G6PD, ACACA, PHGDH, IDH2, AKR1A1, MET, and BCL2L11), the relative transcription abundances were calculated as RTA = E^ΔCt(control-sample)^_(Target)_ / E^ΔCt(control-sample)^_(Reference)_ [129] where control refers to a mix of equal amounts of untreated samples. Fold changes were calculated as the ratio between RTA values obtained for PE5 treated cells and those obtained for untreated cells. The absence of genomic DNA contamination was checked using non-retrotranscriptase controls and the absence of environmental contamination using non-template controls.

## CONCLUSION

During the past decade, cancer metabolism has emerged as one of the most exciting and promising fields for the development of selective anticancer therapies. The development of cancer metabolism inhibitors and their synergy with traditional therapeutic approaches may represent a fundamental change and a promising strategy to overcome resistance in cancer therapy, a phenomenon that unfortunately is cause of treatment failure and recidivism in cancer patients. However, new anticancer drugs have to tackle with the multifactorial phenotype of tumor cells. PE5 significantly inhibits genes that belong to metabolic pathways deregulated in cancer cells and it has the plus of presenting pleiotropic effects. Thus, ND-RNases are very interesting antitumor agents because they can cope with the complex cancer cell phenotype. In addition, their multiple effects allow anticipating synergism with many at present antitumor drugs used in clinical.

## SUPPLEMENTARY TABLES AND FIGURES


