# Supplementary material for: A nuclear-directed human pancreatic ribonuclease (PE5) targets the metabolic phenotype of cancer cells
Source: Oncotarget. 2016 Feb 22;7(14):18309–24. doi: 10.18632/oncotarget.7579 (PMC4951290; doi:10.18632/oncotarget.7579)
Supplement: Supplementary file 1 [file oncotarget-07-18309-s001.pdf]

SUPPLEMENTARY TABLES AND FIGURES

Supplementary Table S1: PE5 differentially expressed genes in NCI/ADR-RES cell line: top 20 PE5 up-regulated and down-regulated genes

Probe ID	Gene Symbol	Gene Name	Fold Change	Main Functions
A_24_P932736	HMBOX1	Homeobox containing 1	106.0	Transcription regulation
A_33_P3236416	GPR179	G protein-coupled receptor 179	65.9	Sensory transduction
A_23_P155463	LRRC2	Leucine rich repeat containing 2	62.7	Unknown
A_33_P3243405	GPR182	G protein-coupled receptor 182	56.3	Signaling pathway
A_23_P119448	PPP6R1	Protein phosphatase 6, regulatory subunit 1	51.5	Signal transduction
A_19_P00809119	LINC00340	Long intergenic non-protein coding RNA 340	45.7	Transcription regulation
A_24_P131589	CD86	CD86 molecule	45.3	Immune response
A_33_P3347417	SPEN	Spen homolog, transcriptional regulator (Drosophila)	41.8	Transcription regulation
A_33_P3315263	KRT79	Keratin 79	40.1	Cell structural integrity
A_23_P254212	RPA4	Replication protein A4, 30kDa	39.6	Cell cycle<br>DNA repair
A_33_P3293913	BICC1	Bicaudal C homolog 1 (Drosophila)	26.3	Embryonic development
A_24_P18802	VPS18	Vacuolar protein sorting 18 homolog (S. cerevisiae)	24.8	Vesicle trafficking
A_33_P3389827	PROM2	Prominin 2	19.7	Membrane organization
A_33_P3247624	REP15	RAB15 effector protein	16.5	Iron metabolism
A_24_P36890	RAP1GAP	RAP1 GTPase activating protein	13.4	Signal transduction
A_24_P151582	TEF	Thyrotrophic embryonic factor	11.9	Transcription regulation
A_23_P144096	CISH	Cytokine inducible SH2-containing protein	11.7	Signal transduction
A_23_P396981	CCDC66	Coiled-coil domain containing 66	9.5	Embryonic development
A_33_P3359368	DHRS4L1	Dehydrogenase/reductase (SDR family) member 4 like 1	8.9	Unknown
A_23_P66543	PIK3R5	Phosphoinositide-3-kinase, regulatory subunit 5	7.0	Signal transduction
A_23_P58082	CCDC80	Coiled-coil domain containing 80	-2.7	Cell adhesion
A_23_P85783	PHGDH	Phosphoglycerate dehydrogenase	-2.7	Amino acid metabolism
A_24_P935986	BCAT1	Branched chain amino acid transaminase 1, cytosolic	-2.8	Amino acid metabolism<br>Cell cycle
A_33_P3290403	IMPA2	Inositol(myo)-1(or 4)-monophosphatase 2	-2.8	Signal transduction
A_33_P3336700	SHROOM3	Shroom family member 3	-2.8	Cell shape regulation
A_23_P156327	TGFBI	Transforming growth factor, beta-induced, 68kDa	-2.8	Cell adhesion<br>Cell proliferation

(*Continued*)

Probe ID	Gene Symbol	Gene Name	Fold Change	Main Functions
A_24_P100613	LAMA1	Laminin, alpha 1	-2.8	Embryonic development<br>Cell adhesion<br>Cell migration
A_32_P107876	FRAS1	Fraser syndrome 1	-2.8	Embryonic development
A_24_P261417	DKK3	Dickkopf 3 homolog (Xenopus laevis)	-2.8	Embryonic development<br>Transcription regulation
A_32_P524014	UTRN	Utrophin	-2.9	Neuromuscular synapse
A_23_P135548	DPYD	Dihydropyrimidine dehydrogenase	-2.9	Pyrimidine metabolism
A_24_P131522	ANTXR1	Anthrax toxin receptor 1	-2.9	Cell adhesion<br>Cell migration<br>Angiogenesis
A_33_P3271930	PYCR1	Pyrroline-5-carboxylate reductase 1	-3.0	Amino acid metabolism<br>Stress response
A_23_P128817	PCK2	Phosphoenolpyruvate carboxykinase 2 (mitochondrial)	-3.0	Gluconeogenesis
A_24_P99216	LRP10	Low density lipoprotein receptor-related protein 10	-3.1	Lipid metabolism
A_23_P395172	ABHD2	Abhydrolase domain containing 2	-3.1	Cell migration
A_23_P501007	EFEMP1	EGF containing fibulin-like extracellular matrix protein 1	-3.1	Cell adhesion<br>Cell migration<br>Transcription regulation
A_23_P33894	MAGED2	Melanoma antigen family D, 2	-3.1	Signal transduction<br>Apoptosis<br>Cell cycle
A_24_P309317	PSAP	Prosaposin	-3.2	Sphingolipid metabolism
A_32_P97169	GPC6	Glypican 6	-4.1	Cell migration

Gene information was taken from the UniProt database (European Bioinformatics, UK, Swiss Institute of Bioinformatics, Switzerland, Protein Information Resource, USA) (http://www.uniprot.org) and from the Entrez Gene database (National Center for Biotechnology Information, USA) (http://www.ncbi.nlm.nih. gov/gene).

Supplementary Table S2: List of constitutive genes examined by RT-qPCR. To select a constitutive gene as a reference for normalizing data, five genes were analyzed by RT-qPCR

Gene Symbol	Gene Name	Primers used [a]
ACTB	Actin, beta	F: TGGCATCCACGAAACTACCTT<br>R: CAGGGCAGTGATCTCCTTCTG
GUSB	Glucuronidase, beta	F: GAACGCCCTGCCTATCTGTATT<br>R: ATGAGGAACTGGCTCTTGGTG
TBP	TATA box binding protein	F: GGCACCACAGCTCTTCCACT<br>R: TGCGGTACAATCCCAGAACTC
HPRT1	Hypoxanthine phosphoribosyltransferase 1	F: CAGACTTTGCTTTCCTTGGTCA<br>R: AACACTTCGTGGGGTCCTTT
ALAS1	Aminolevulinate, delta-, synthase 1	F: ACCCTCTTCACCCTGGCTAA<br>R: ACTTTGGCACTCGGCTGTTT

[a] F: Forward primer
R: Reverse primer

Supplementary Table S3: List of target genes examined by RT-qPCR.

Biological process	Gene Symbol	Gene Name	Primers used [a]
Carbohydrate metabolism	G6PD	Glucose-6-phosphate dehydrogenase	F: TGGAGAATGAGAGGTGGGATG<br>R: GCACTGCTGGTGGAAGATGTC
Lipid metabolism	ACACA	Acetyl-CoA carboxylase alpha	F: TACAACGCAGGCATCAGAAGA<br>R: CAGCACTCACATAACCCACCAT
Amino acid metabolism	PHGDH	Phosphoglycerate dehydrogenase	F: TATTGTTCGCTCTGCCACCA<br>R: TCATAACCAAGATGCCCTTCC
Quenching of ROS/ TCA cycle	IDH2	Isocitrate dehydrogenase 2 ($NADP^+$), mitochondrial	F: GTCTTCGGGTGGCTTTGTGT<br>R: CCTCAATCGTCTTCCCATCAG
Drug resistance	AKR1A1	Aldo-keto reductase family 1, member A1 (aldehyde reductase)	F: CCTGGAAGAGTGAGCCTGGT<br>R: ACAATCAATGTGGCGGTAGC
Oncogenes	MET	Met proto-oncogene (hepatocyte growth factor receptor)	F: ATCCTCGTGCTCCTGTTTACCT<br>R: CACATTCATCTCGGACTTTGCT
Tumor suppressors	BCL2L11	BCL2-like 11 (apoptosis facilitator)	F: GCAACCTTCTGATGTAAGTTCTGA<br>R: GCTCCTGTCTTGTGGCTCTGT

Seven genes representative of the most interesting biological processes affected by PE5 (carbohydrate, lipid and amino acid metabolism, quenching of reactive oxygen species (ROS)/TCA cycle, drug resistance, oncogenes, and tumor suppressors) were selected to perform RT-qPCR.

[a] F: Forward primer

R: Reverse primer

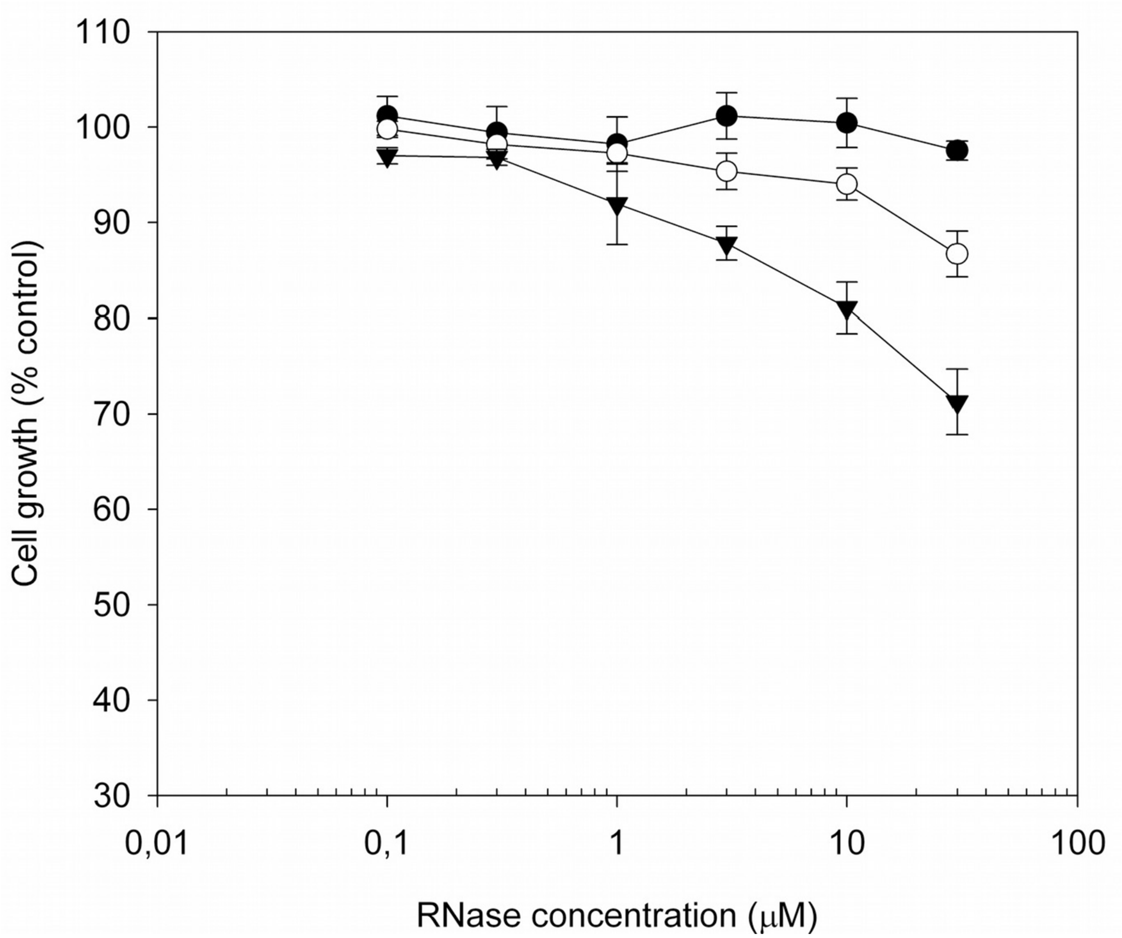

Supplementary Figure S1: Cytotoxic effects of PE5 in NCI/ADR-RES cell line. Control and RNase treated cells were maintained for 24 (●), 36 (○), or 48 h (▼) and metabolic activity was determined by the MTT assay. Cell growth is expressed as the percentage of activity respective to control cells using the absorbance values. The curves in the figure are from one representative experiment made in triplicates. Data are presented as mean ± SD. Equivalent results were found in at least three independent experiments.

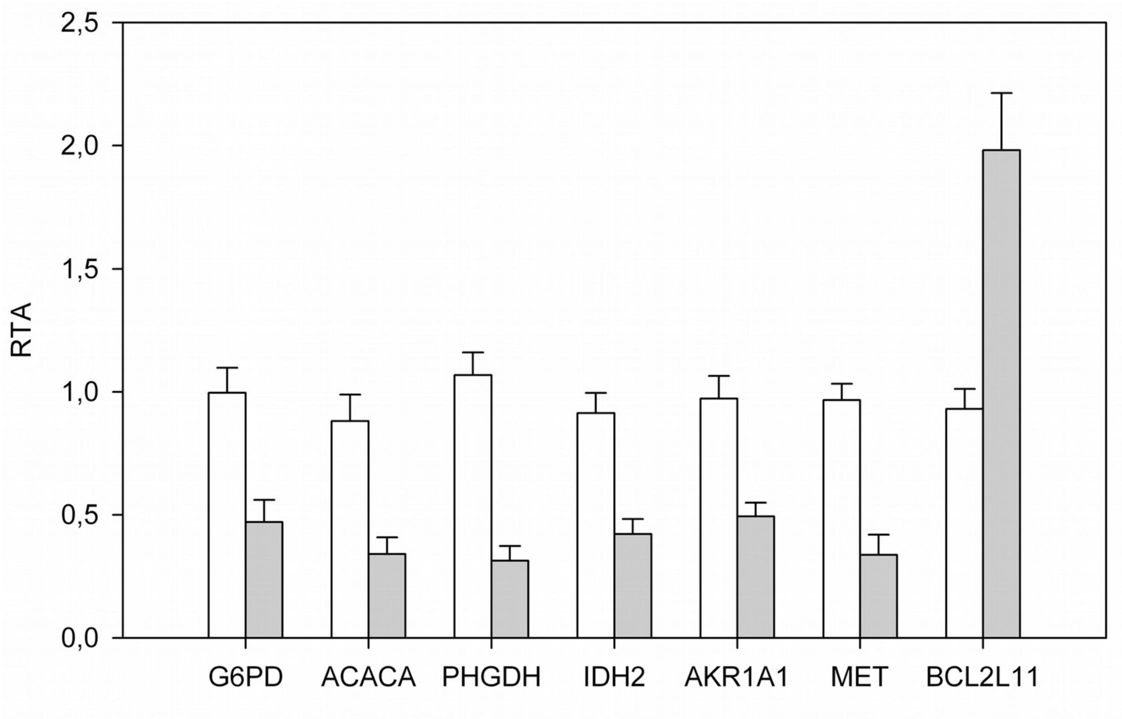

Supplementary Figure S2: Quantitative gene expression changes in NCI-ADR-RES cell line. The histogram shows the relative transcript abundance (RTA) obtained by RT-qPCR of selected genes up or down-regulated by PE5. White bars, untreated cells; grey bars, PE5-treated cells. Data are presented as mean ± SD. Genes: Glucose-6-phosphate dehydrogenase (G6PD), Acetyl-CoA carboxylase alpha (ACACA), Phosphoglycerate dehydrogenase (PHGDH), Isocitrate dehydrogenase 2 ($NADP^+$), mitochondrial (IDH2), Aldo-keto reductase family 1, member A1 (aldehyde reductase) (AKR1A1), Met proto-oncogene (hepatocyte growth factor) (MET), and BCL2-like 11 (apoptosis facilitator) (BCL2L11).

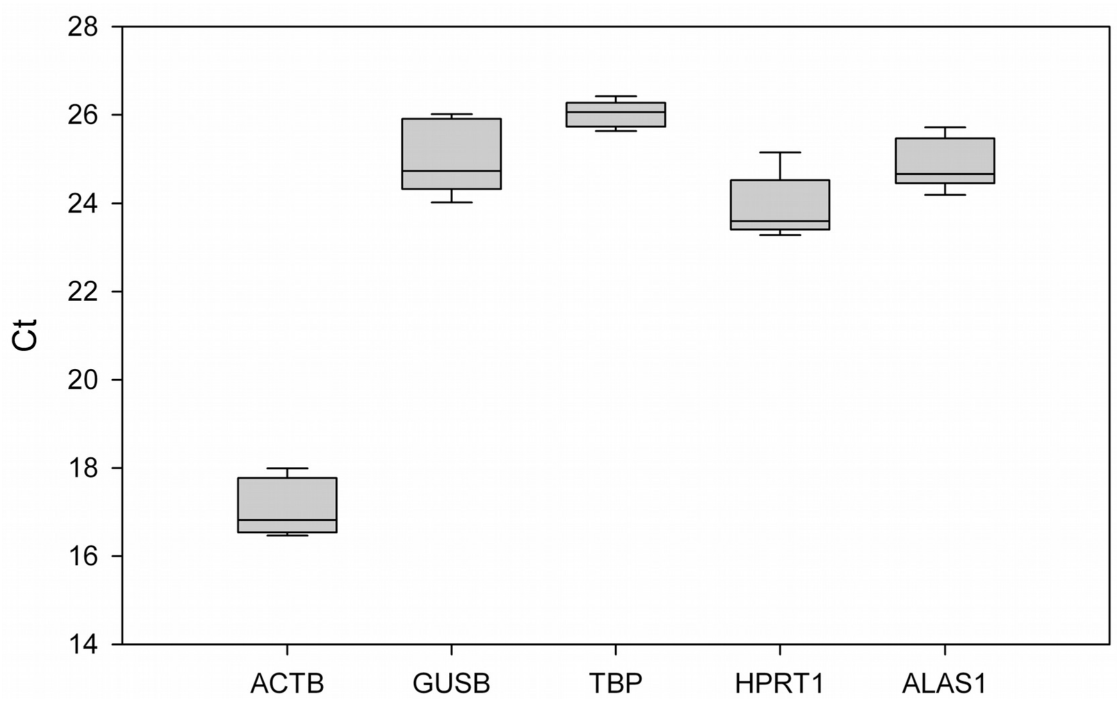

Supplementary Figure S3: Validation of constitutive genes. Box plot of the threshold cycle (Ct) values of five constitutive genes: actin beta (ACTB), glucuronidase beta (GUSB), TATA box binding protein (TBP), hypoxanthine phosphoribosyltransferase 1 (HPRT1) and 5'-aminolevulinate synthase 1 (ALAS1). To select a reference for normalizing RT-qPCR data, the transcription abundances of these genes were measured for all cDNA samples. Among them, TBP showed the highest stability (lower standard deviation of the Ct) and was chosen to normalize the RT-qPCR results.
